# Effects of Non-verbal Priming on Attachment-Style Activation

**DOI:** 10.3389/fpsyg.2019.00684

**Published:** 2019-04-05

**Authors:** Suhyung Sim, Ji-eun Shin, Young Woo Sohn

**Affiliations:** ^1^Department of Psychology, Yonsei University, Seoul, South Korea; ^2^Department of Psychology, Chonnam National University, Gwangju, South Korea

**Keywords:** non-verbal cues, attachment style, affective priming, self-esteem, interpersonal competency, positive affect

## Abstract

Using an affective priming procedure, two experiments examined the effects of non-verbal cues on activating attachment styles. In Study 1, the secure attachment group, which was primed non-verbally, showed higher levels of self-esteem, interpersonal competence, and positive affect than the insecure attachment group, which was also primed non-verbally. In contrast, no significant difference was found between the two attachment groups that were primed verbally. In Study 2, using a different priming method and adding a neutral group, similar interactions between priming modality (non-verbal or verbal cues) and attachment styles were found: the differences in self-esteem, interpersonal competence, and positive affect between the secure attachment group, neutral group, and insecure attachment group were greater when primed non-verbally than when primed verbally. Finally, the limitations of the research and recommendations for follow-up study are discussed.

## Introduction

We communicate with others everyday using numerous verbal and non-verbal cues. Words are the most effective means for conveying messages that contain factual information (Chaiken and Eagly, 1976), but this may not be the case for emotional messages. In an attempt to offer consolation to distressed others, for example, a simple touch on the shoulder can be more effective than words (Ditzen et al., 2007). This is because emotion tends to be expressed and perceived through non-verbal channels, such as facial expression, voice tone, and posture, rather than verbally (Ekman and Friesen, 1975; Pally, 2008; App et al., 2011). Moreover, social communication—such as knowing what another is feeling and how one should respond to that emotional state—precedes the evolution of verbal abilities (Darwin, 1872/1998); thus it is not surprising that people communicate emotions through several non-verbal channels, including the face, body, and touch (Buck, 1984; App et al., 2011). In addition, unlike the verbal communication that occurs only through language, an emotion is a useful reaction that quickly transmits affective meaning in a natural way through the non-verbal channels noted above (Vrij, 2004; Weisbuch and Ambady, 2008). Based on the role non-verbal cues play in emotional communication, we aimed to examine how non-verbal cues relate to the fundamental basis of human emotional development: attachment (Thompson, 1994; Tucker and Anders, 1998; Riggio, 2006).

According to attachment theory, humans form attachment representations of self and others through interactions with primary caregivers (Ainsworth, 1969; Bowlby, 1969, 1973; Mahler et al., 1975; Fonagy, 2001). Here, “attachment representation” influences how people interpret their own behavior and feelings, as well as those of others (Beijersbergen et al., 2012). Attachment styles can generally be classified in two ways: secure attachment and insecure attachment (avoidant attachment and anxious attachment; Ainsworth et al., 1978/2015). Several studies have shown that securely attached individuals are likely to hold positive views of themselves and others, are more confident in their ability to deal with threats and challenges, and tend to employ more constructive and effective strategies for emotion regulation (e.g., problem solving, reappraisal, support seeking). Moreover, secure people can remain open to their emotions, express and communicate feelings freely and accurately to others, and experience them fully without distortion (Mikulincer and Shaver, 2018). On the other hand, insecurely attached people suppress their negative emotions and continue to rely on distorted views of self and others, which contributes to poor physical and mental health (Mikulincer and Shaver, 2016). Attachment anxiety triggers worries that others will not be available in times of need, and attachment avoidance gives rise to compulsive self-reliance and distrust of others’ intentions. Both become maladaptive when applied to adult relationships in which support seeking and comfortable interdependence can help a person maintain a sense of well-being (Shaver and Mikulincer, 2002).

Attachment styles are largely based on attachment experiences in childhood and adolescence, and continue to exert an influence in adulthood in contexts that activate the attachment system, such as a romantic partner or close friends (Hazan and Zeifman, 1994; Ravitz et al., 2010). For instance, greater insecurity regarding parents and peers in adolescence predicts a more anxious romantic attachment style and greater use of emotion-oriented strategies (e.g., worry about what action one should take) in adulthood (Pascuzzo et al., 2013). Indeed, activated mental representations of attachment figures (e.g., subliminal presentation of their names) result in heightened security and reduced hostility to out-group members, facilitate compassion and altruistic behavior toward needy others, and sustain creative problem solving (Mikulincer and Shaver, 2012).

In the formation of attachment representation, a focus on non-verbal behavior has played a substantial role (Mahler et al., 1975). Empirical evidence has established that the foundations of our mental representations are in place well before the acquisition of language (Wallin, 2007). Specifically, children develop an attachment style in the preverbal stage when they communicate with their primary caregiver through non-verbal cues. When an infant cries, the mother feeds it. When a baby is scared, the mother holds and reassures it. Such ordinary, repeated experiences confer a sense of secure attachment: “I’m a good enough person and people are trustworthy enough to be relied on” (Bowlby, 1969, 1973).

Nevertheless, few studies have investigated the non-verbal aspects of attachment style. Since non-verbal cues are important in the formation of an attachment style, they may still be important when the attachment representation is activated in adulthood. For instance, one can become calm and obtain a sense of security by simply visualizing the attachment figure’s face (Mikulincer and Shaver, 2004; Mikulincer et al., 2005). Specifically, Jakubiak and Feeney (2016b) found that individuals who are reminded of affective experiences in which their attachment figure supported them using touch experienced less stress and were more willing to accept a challenge than those who were reminded of experiences in which their attachment figure supported them using words. This implies that there are two channels, non-verbal and verbal, through which an attachment style can be activated, and that the influence of each channel may vary. Jakubiak and Feeney’s results show that the non-verbal channel is superior for conveying affective meaning. Wallin (2007) supports this argument by demonstrating that attachment representation cannot be linguistically retrieved, because our first relational experiences are mainly outside the domain of language. Therefore, we focus on the operating characteristics of the non-verbal channel in terms of attachment representation.

Further, previous studies that examined non-verbal behaviors and attachment style are limited, in that they do not consider the entire spectrum of non-verbal modalities (Scherer, 1995; Mikulincer and Shaver, 2007; Xiao et al., 2016). Non-verbal behaviors do not function independent of each other, but instead involve various behaviors that operate in multiple ways through different routes (Gunes et al., 2008). Accordingly, previous studies that focus only on one non-verbal modality, such as touch (Jakubiak and Feeney, 2016a), vocal rhythm (Beebe et al., 2000), or facial expression (Niedenthal et al., 2002) provide valuable information about the properties of a given non-verbal cue, but offer no comprehensive guidance about the effects of non-verbal behavior on an attachment style.

### The Present Study

This study aimed to determine whether non-verbal behavior is an important factor in activating an attachment style. Specifically, in Study 1, we used an affective priming technique to examine whether a receptive non-verbal behavior and a rejecting non-verbal behavior of a significant other (e.g., primary caregiver) activate secure and insecure attachments, respectively. If one repeatedly experiences a significant other as being at one’s side, responsive, and supportive, secure attachment is formed. Thus, we expected that a secure attachment style would be activated for a group that is reminded of a scene in which a significant other warmly gazes at them and supports them with non-verbal cues, and that an insecure attachment style would be activated for a group that is reminded of a scene in which a significant other discourages or rejects them with non-verbal cues. A verbal priming condition was used with the control group, which was instructed to recollect an experience of being accepted by or an experience of being rejected by the significant other. In addition, the priority was to recall what words had been said to them. This condition distinguishes this study and previous attachment studies, because we divided attachment-style activation routes into non-verbal and verbal priming and examined them under the same experimental conditions to compare variations according to different routes.

Specifically, we compared the magnitudes of the difference between secure and insecure attachments activated by non-verbal priming and the difference between secure and insecure attachments activated by verbal priming. For this purpose, we measured self-esteem, interpersonal competence, and positive affect through self-report questionnaires because the greater differences between secure and insecure attachment styles were revealed mainly in three areas: self-worth, confidence in others, and affective aspects (Feeney and Kirkpatrick, 1996; Fraley and Shaver, 1998; Collins and Feeney, 2000; Mikulincer et al., 2002).

Taking these individually, first, a person with a secure attachment style has high self-esteem (Mickelson et al., 1997), confidence in one’s own abilities, and the ability to use effective emotion regulation strategies in stressful situations (Canterberry and Gillath, 2013). Second, a person with a secure attachment style has confidence in others and expects their goodwill. Accordingly, the person does not become unnecessarily discouraged in interpersonal relationships and shows a high level of sociality (Hazan and Shaver, 1990; Mikulincer et al., 2001). Third, a person with a secure attachment style is more positive about life and experiences positive emotions more often (Selcuk et al., 2012). A psychological effect that can be immediately obtained from an attachment figure is affective stability, because one’s interaction with an accessible and supportive attachment figure transfers that person’s sense of security to oneself, thereby reducing one’s anxiety and awakening positive emotions such as relief, satisfaction, and appreciation (Mikulincer and Shaver, 2007). For an infant to gain a sense of security, he or she must be approached by, see, and touch the attachment figure. In adulthood, however, other attachment strategies are available. For instance, a romantic partner or close friend can be an attachment figure, and one can become calm and obtain a sense of security by simply visualizing one’s experiences with such a figure (Mikulincer and Shaver, 2004).

In Study 2, in line with Study 1, we used an affective priming technique to examine whether a non-verbal cue activates secure and insecure attachments. In this case, however, we aimed to improve the reliability and validity of the experiment through the use of a different attachment priming method, a larger sample size and the addition of a neutral group. Among these, the most critical difference between Studies 1 and 2 was the priming method. As described above for Study 1, through the scenario priming method, the focus was on activating attachment based on the non-verbal cue (i.e., eyes, posture, silence, etc.) or the verbal cue of the significant other. In contrast, in Study 2, we focused on priming attachment through the exclusive use of non-verbal or verbal cues. More specifically, in the case of the non-verbal priming condition, we used attachment-related photographs, which can be classified as non-verbal stimuli. In the case of the verbal priming condition, we used attachment-related words that can be regarded as verbal stimuli. Many studies have primed secure attachment by using such non-verbal stimuli as exposing participants to pictures that represent secure attachment (Zayas and Shoda, 2005; Mikulincer and Shaver, 2007; Bowles and Meyer, 2008; He et al., 2011). Typically, the black-and white Picasso sketch that depicts a mother holding and looking at her baby (Mikulincer et al., 2001) or attachment-related images such as photographs of a mother holding a baby are used (Bryant and Foord, 2016). In line with these previous studies, photographs that prime secure and insecure attachments were used for the non-verbally primed group in this study. For the verbally primed group, texts that explain and describe the photographs were used. This method is typically used in research that identifies differences between non-verbal and verbal cues (Peristeri et al., 2018; Radman et al., 2018). For Studies 1 and 2, we expected that the differences in self-esteem, interpersonal competence, and positive affect between secure and insecure attachment styles that had been primed by non-verbal cues would be greater than the differences between the two styles of attachment primed verbally.

## Study 1

In Study 1, secure and insecure attachment styles were activated by non-verbal priming and verbal priming, and differences in self-esteem, interpersonal competence, and positive affect according to the activated attachment style were examined. Based on previous literature (Anders and Tucker, 2000), secure attachment is presumed to contain the following two core representations: (1) positive representation of oneself (e.g., “I must be a pretty good person; that must be why that a person is consistently interested in me and kind to me”) and (2) positive representation of others (e.g., “People are trustworthy”). In other words, people with secure attachment are likely to have higher self-esteem and view others in a more positive light. They are also expected to be better at forming interpersonal relationships and experience positive emotions more often than people with insecure attachment (Levy et al., 1998).

However, given the importance of non-verbal interaction in attachment formation at the preverbal stage, non-verbal cues may have greater influence than verbal cues on multiple consequences and correlates of attachment (e.g., self-esteem, interpersonal competence, and positive affect). Accordingly, we sought to activate secure and insecure attachments through two priming conditions—non-verbal priming and verbal priming—and to examine the different impacts according to priming modality and attachment style. More specifically, we expected that the differences in self-esteem, interpersonal competence, and positive affect between the secure attachment group and the insecure attachment group would be greater when primed by non-verbal rather than verbal cues.

### Methods

#### Participants

One hundred and seven undergraduates participated for course credit. Twelve participants who did not complete the questionnaire or failed to be primed were excluded, leaving a total of 93 participants (27 men, 66 women). The mean age was 22.4 years, ranging from 19 to 29 years (*SD* = 1.84).

#### Experimental Design

We employed a 2 (priming modality: non-verbal vs. verbal) × 2 (attachment style: secure vs. insecure) factorial design. Participants were randomly assigned to one of four groups: secure attachment through non-verbal cues (*n* = 25), insecure attachment through non-verbal cues (*n* = 26), secure attachment through verbal cues (*n* = 22), and insecure attachment through verbal cues (*n* = 20). Our dependent variables were self-esteem, interpersonal competence, and positive affect.

#### Measurement Tools

##### Scenario priming

A sense of acceptance is considered to be a core element of attachment representation (Sarason et al., 1990). Based on this, we chose the contents of the scenarios for secure and insecure attachment styles. According to the sensory modality priming condition, the relative weights of the non-verbal and verbal cues of the significant other were assigned differently. Specifically, participants in the secure attachment condition were instructed to recall and describe an instance in which they were with a significant other who tends to be accepting and non-evaluative of them. They were also directed to recollect in detail what the non-verbal cues of the person were at that moment. In contrast, participants in the insecure attachment condition were instructed to recall and describe a moment when they were non-verbally rejected and discouraged by a significant other. For groups with secure and insecure attachments primed by verbal cues of the significant other, **i**nstructions for the non-verbal cue portion were changed to “recollect the content of his or her words in detail.”^1^ By using guided imagery, albeit with differential priming through non-verbal or verbal cues of significant others for each group, we followed the standard research method employed in previous studies (Jakubiak and Feeney, 2016b; see Appendix).

##### Self-esteem

The Single-Item Self-Esteem Scale (Robins et al., 2001) was used to measure self-worth. A sample question is, “At this moment, I have high self-esteem,” and responses were assessed on a five-point Likert scale (1 = *Not at all*, 5 = *Strongly agree*). The scale was chosen as an alternative to using the Rosenberg Self-Esteem Scale, which has a 10-item scale. Though shorter, the scale has strong convergent validity with the Rosenberg Self-Esteem Scale, as well as similar predictive validity (Robins et al., 2001).

##### Interpersonal competence

We used the Interpersonal Competence Questionnaire Scale (Buhrmester et al., 1988), which contains 31 items and five subscales on initiating relationships, self-disclosure, asserting displeasure with others’ actions, providing emotional support, and managing interpersonal conflicts. Individual items are measured on a five-point Likert scale (1 = *Not at all*, 5 = *Strongly disagree*). Scores on the five subscales (α = 0.86) were summed to obtain an overall score; a higher score indicates greater interpersonal competence.

##### Positive affect

From the Scale of Positive and Negative Experience developed by Diener et al. (2009) to measure positive and negative experiences, we used six items that measure positive experience. A sample item is, “Please circle how much you experience each of the following feelings or emotions right now at this moment,” followed by six adjectives (joyful, happy, positive, contented, pleasant, and delighted). To capture subtle differences, the original five-point Likert scale for each item was expanded to a seven-point scale (1 = *Very rarely or never*, 7 = *Very often or always*). The scores of all items were summed (α = 0.95), with a higher score indicating greater positive affect.

#### Procedure

After completing the informed consent form, each participant was taken to a separate space divided by partitions. Participants were randomly assigned to four groups, and corresponding scenarios were presented to each group. They were given 6 min to read the scenario and write about their recalled memories, after which they responded to questions about whether their recalled memories and emotions were (1) accurately recollected, (2) easily recollected, or (3) vividly called to mind even now, using a seven-point Likert scale (1 = *Not at all*, 7 = *Extremely*; Baldwin and Sinclair, 1996). For a manipulation check, we only included data from participants who scored at least four points on three questions. Two raters read and evaluated participants’ recalled memories. If their ratings did not coincide with each other, it was also regarded as a priming failure. After the priming manipulation, participants completed a questionnaire.

### Results

**Figure 1:**
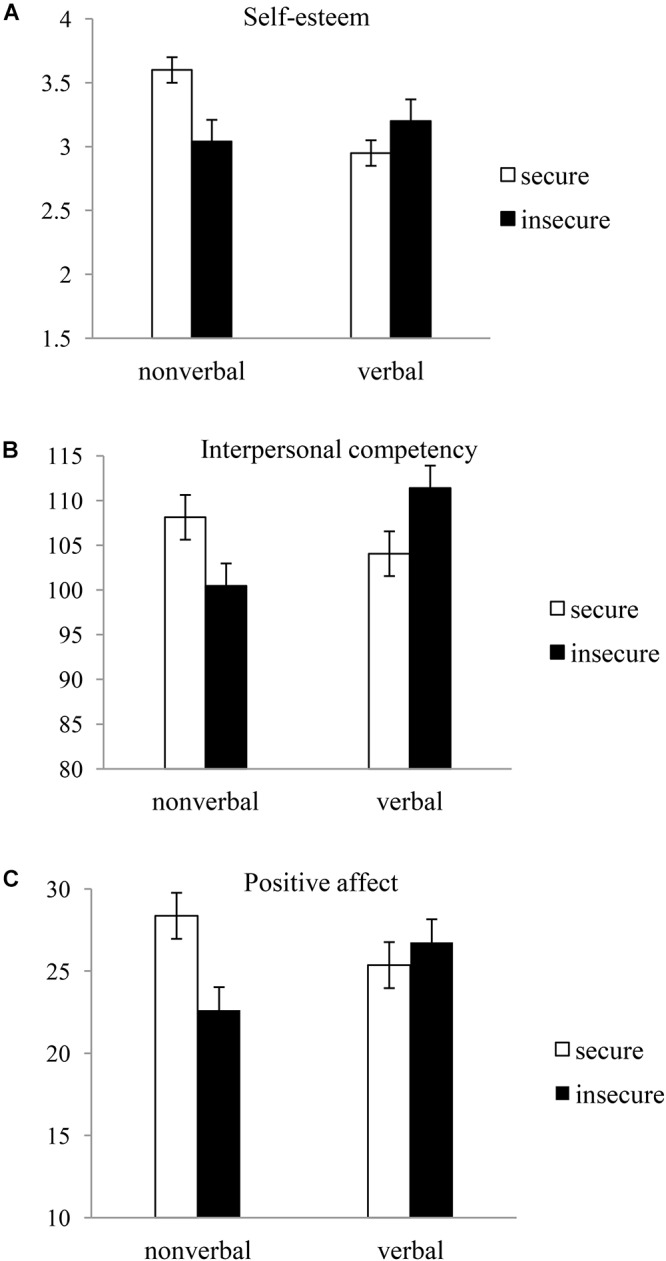
Mean scores of **(A)** self-esteem, **(B)** interpersonal competency, and **(C)** positive affect against priming modality and primed attachment style (Study 1).

We compared differences in self-esteem, interpersonal competence, and positive affect between the two groups that had activated secure and insecure attachments through non-verbal cues with the corresponding differences between the two groups that had activated secure and insecure attachments through verbal cues. For the purpose of this comparison, we performed a 2 (priming modality: non-verbal vs. verbal) × 2 (attachment style: secure vs. insecure) analysis of variance. Both factors were between-subject variables. Figure 1 shows the means and standard errors for each dependent variable as a function of priming modality and attachment style.

#### Self-Esteem

The 2 × 2 ANOVA for self-esteem showed that there were no main effects of priming modality, *F* < 1.97, *p* = 0.17, η^2^ = 0.02, and attachment style, *F* < 1, *p* = 0.36, η^2^ = 0.01. As expected, however, there was a significant interaction between priming modality and attachment style, *F*(1,91) = 5.45, *p* = 0.02, η^2^ = 0.06. As shown in Figure 1A, a significant difference in self-esteem was found between secure and insecure attachment groups primed by non-verbal cues, *t*(51) = 2.58, *p* = 0.01. More specifically, participants in the group in which secure attachment was primed by non-verbal cues (*M* = 3.60, *SD* = 0.86) showed greater self-esteem than those in the group in which insecure attachment was primed (*M* = 3.04, *SD* = 0.70). Conversely, there was no significant difference between the two attachment styles primed by verbal cues, *t*(41) = -1.07, *p* = 0.29.

### Interpersonal Competence

The 2 × 2 ANOVA for interpersonal competence showed that there were no main effects of priming modality, *F* < 1.8, *p* = 0.19, η^2^ = 0.02, and attachment style, *F* < 1, *p* = 0.95, η^2^ = 0.00. However, the interaction between priming modality and attachment style was significant, *F*(1,89) = 8.29, *p* = 0.01, η^2^ = 0.09. As shown in Figure 1B, there was a significant difference in interpersonal competence between the two attachment styles primed by non-verbal cues, *t*(50) = 2.11, *p* = 0.04. More specifically, participants in the group in which secure attachment was primed by non-verbal cues (*M* = 108.12, *SD* = 13.00) showed greater interpersonal competence than those in the group in which insecure attachment was primed (*M* = 100.46, *SD* = 12.90). On the other hand, there was no significant difference between the two attachment styles primed by verbal cues, *t*(41) = -1.99, *p* = 0.06.

### Positive Affect

The 2 × 2 ANOVA for positive affect showed that there were no main effects of priming modality, *F* < 1, *p* = 0.70, η^2^ = 0.00, and attachment style, *F* < 2.5, *p* = 0.14, η^2^ = 0.02. However, the interaction between priming modality and attachment style was significant, *F*(1,91) = 5.83, *p* = 0.02, η^2^ = 0.6. As shown in Figure 1C, for the non-verbally primed condition, the secure attachment group (*M* = 28.36, *SD* = 6.31) showed greater positive affect than the insecure attachment group [*M* = 22.62, *SD* = 7.56), *t*(51) = 2.80, *p* = 0.01]. Conversely, there was no significant difference between the two verbally primed attachment styles, *t*(41) = -0.3, *p* = 0.77.

In summary, for the non-verbal priming condition, participants primed with secure attachment showed greater self-esteem, interpersonal competence, and positive affect than those primed with insecure attachment. On the other hand, no significant differences were found for the verbal priming condition. This suggests that the consequences of each attachment style are more apparent when it is activated by non-verbal than verbal cues.

However, this study has several limitations. First, the sample size was not sufficiently large. Second, each participant’s attachment orientation was not measured. However, previous research has shown that participants’ original attachment orientation is independent of the style of attachment primed (Carnelley and Rowe, 2007; Gillath et al., 2010; Luke et al., 2012). That is, our priming manipulations are based on the fact that even though people have an attachment orientation, most can change their attachment orientation over time and are sensitive to specific situations (Cozzarelli et al., 2003; Birnbaum et al., 2012; Hudson, 2016). If we compared participants’ attachment orientation, however, we could determine whether it moderates the effects of the attachment primed. Third, the priming method might not be effective for activating non-verbal or verbal cues exclusively. Although we asked participants to concentrate on a significant other’s non-verbal or verbal cues, there is a possibility that participants mixed the non-verbal and verbal cues when recollecting the memory of interaction with the significant other. Lastly, because we did not include a neutral condition, it is difficult to clearly determine the priming effects. If each of the primed attachment groups were compared to a neutral group, we could obtain a more valid test of the unique effects of securely and insecurely primed attachments. Accordingly, Study 2 aimed to close these gaps.

## Study 2

As in Study 1, the main goals of Study 2 were to examine whether secure and insecure attachments are activated through non-verbal and verbal priming, and whether differences in self-esteem, interpersonal competence, and positive affect arose from the attachment style activated. To overcome the limitations of Study 1, we increased sample sizes and measured participants’ attachment orientation. Also, for the non-verbal condition, photographs were used as representative, non-verbal stimuli. For the verbal condition, we used text that described each photograph. Finally, we included an attachment-neutral condition to examine the differences between the primed attachment groups and the neutral group. We anticipated that the differences in self-esteem, interpersonal competence, and positive affect between the secure and insecure attachment groups would be greater when the priming took place non-verbally than verbally.

### Methods

#### Participants

Four hundred nine people participated in the online experiment. Because 10 participants answered the questionnaire insincerely or failed the priming, they were excluded from the analysis. Data for 399 participants (141 males, 258 females) were used. The mean age was 38.3 years, ranging from 19 to 49 (*SD* = 8.25).

#### Experimental Design

We employed a 2 (priming modality: non-verbal vs. verbal) × 3 (attachment style: secure vs. insecure vs. neutral) factorial design. Participants were randomly assigned to one of six groups: secure attachment through non-verbal cues (*n* = 64), insecure attachment through non-verbal cues (*n* = 65), neutral through non-verbal cues (*n* = 68), secure attachment through verbal cues (*n* = 68), insecure attachment through verbal cues (*n* = 67), and neutral through verbal cues (*n* = 67). As dependent variables, self-esteem, interpersonal competence, and positive affect were measured in the same way as in Study 1.

#### Measurement Tools

##### Non-verbal/verbal priming

Based on the priming condition, photographs or words were presented in the middle of the computer screen. For the condition in which secure attachment was primed nonverbally, the photograph of a mother feeding her baby as they looked at each other was presented. For the condition in which insecure attachment was primed nonverbally, a photograph of a male holding a baseball bat and a girl curled up on the ground was presented. For the neutral nonverbal condition, a photograph of 12 interconnected gray heptagons was presented. In contrast, descriptions of the photographs were presented for the verbal priming conditions as follows: “A mother wearing a white shirt and white pants is sitting on a white mattress and feeding her baby boy, who is wearing diapers as they look at each other and smile.” for the secure attachment condition, “A strong man’s lower body is viewed from the rear. He is wearing white pants and holding a baseball bat in his right hand, and a girl who is wearing a white top and short blue pants is curled up on the floor in a corner.” for the insecure attachment condition, and “Twelve overlapping gray heptagons are presented against a gray background.” for the neutral condition. For all the conditions, we chose stimuli that are commonly used in research on attachment (Dalgleish et al., 2007; Li and Kato, 2011).

##### Self-esteem, interpersonal competence, and positive affect

The same procedures were used as in Study 1. The internal consistency of interpersonal competence was α = 0.93, and the scale of positive affect was α = 0.97.

##### Adult attachment style

To measure each participant’s attachment orientation, the Experiences in Close Relationships-Revised Questionnaire (ECR-R; Fraley et al., 2000) was used. The scale contains 36 items and is divided into two subscales: anxiety attachment (α = 0.92), which represents rejection or abandonment by others, and avoidance attachment (α = 0.87), which measures discomfort related to becoming intimate with and relying on another person. The higher the total score (α = 0.90), the greater the anxiety and avoidance triggered by attachment. Each item was rated on a five-point Likert scale (1 = *Not at all* to 5 = *Strongly agree*). A sample avoidance item is, “I prefer not to show others how I feel deep down”; a sample anxiety item is, “I worry about being rejected or abandoned.”

#### Procedure

After completing the informed consent form, participants were presented with their respective group’s photograph or words and asked to reflect on the following questions: (1) “At that moment, what is child feeling?”; (2) “What kind of a person will the child become in the future, and what kind of life will he or she have?” (Bowles and Meyer, 2008); and (3) “If I were that child, what emotion would I feel at that moment?” While looking at the photograph or reading the words, participants were asked to imagine the situation as deeply as possible. Participants in the neutral group were asked to observe or try to imagine the figures in as much detail as possible. Next, the secure and insecure attachment groups answered questions regarding the clarity, ease, and realism of their recalled memories and emotions on a seven-point Likert scale (1 = *Not at all*, 7 = *Extremely*; Baldwin and Sinclair, 1996). After this, participants completed a questionnaire that measures self-esteem, interpersonal competence, and positive affect. Lastly, to eliminate the priming effect and diffuse any feelings of attention, participants were asked to solve 10 arithmetic problems (e.g., 27+15, 66-7). Afterward, each participant’s attachment orientation was measured.

### Results

**Figure 2:**
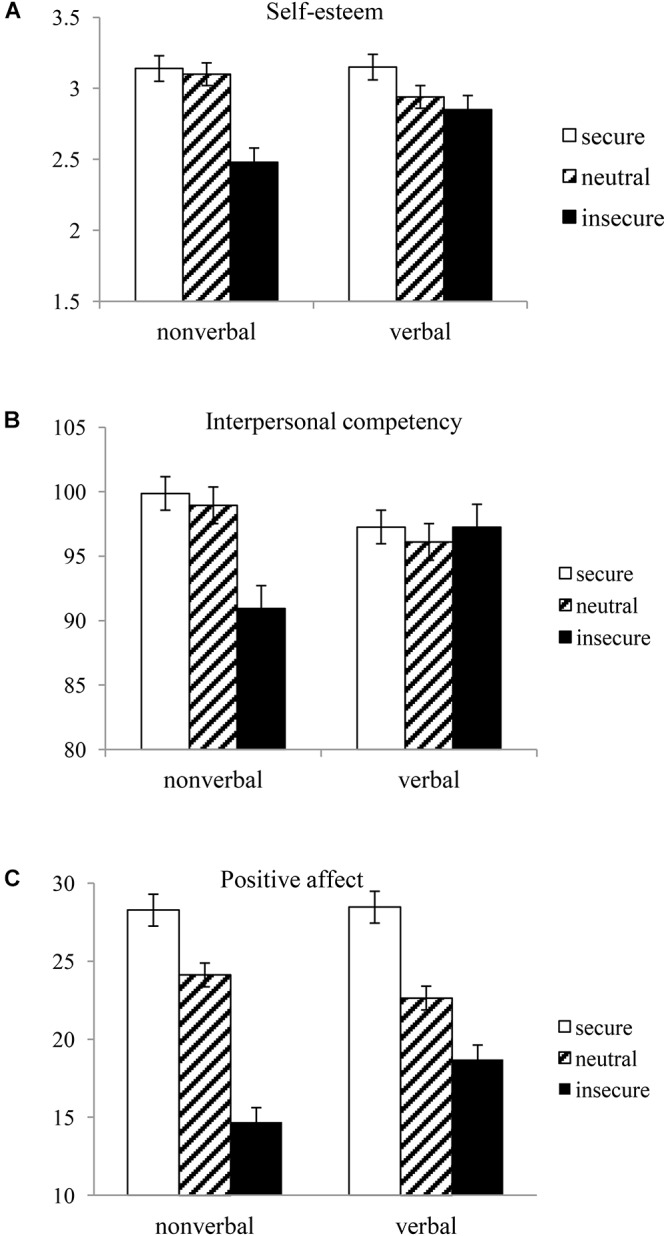
Mean scores of **(A)** self-esteem, **(B)** interpersonal competency, and **(C)** positive affect against priming modality and primed attachment style (Study 2).

We compared differences in self-esteem, interpersonal competence, and positive affect between (a) the three groups in which secure attachment, insecure attachment, and a neutral condition were activated with non-verbal cues and (b) the three corresponding groups activated with verbal cues. For the purpose of this comparison, we performed a 2 (priming modality: non-verbal vs. verbal) × 3 (attachment style: secure vs. insecure vs. neutral) ANOVA on each of the dependent variables—self-esteem, interpersonal competence, and positive affect. Both factors were between-subject variables. Prior to the analysis, we examined the homogeneity of participants in the six groups in terms of their attachment orientation scores, which were found to not vary across all of the six groups, *F*(5,393) = 0.33, *p* = 0.89. In addition, an ANOVA was performed, controlling for attachment orientation scores as the covariates. Figure 2 shows the means and standard errors for each dependent variable as a function of priming modality and attachment style.

#### Self-Esteem

The 2 × 3 ANCOVA for self-esteem showed that the main effect of attachment style was significant, *F*(1,392) = *13.91, p* < 0.00, η^2^ = 0.07, but the main effect of priming modality was not, *F* < 1.03, *p* = 0.31, η^2^ = 0.00. Self-esteem was greater in the following order: secure attachment group (*M* = 3.14, *SD* = 0.79), neutral group (*M* = 3.02*, SD* = 0.72), and insecure attachment group (*M* = 2.67, *SD* = 0.90). As expected, there was a significant interaction between priming modality and attachment style, *F*(2,392) = 4.33, *p* = 0.01, η^2^ = 0.02, which is the crucial part of our analysis in Study 2. As displayed in Figure 2A, a Bonferroni *post hoc* analysis showed that self-esteem was greater for the group in which secure attachment was primed non-verbally (*M* = 3.14, *SD* = 0.79) than for the group in which insecure attachment was primed non-verbally (*M* = 2.48, *SD* = 0.88), *p* = 0.00. Also, self-esteem was greater for the non-verbal neutral group (*M* = 3.10, *SD* = 0.75) than for the group in which insecure attachment was primed non-verbally (*M* = 2.48, *SD* = 0.88), *p* = 0.00. In contrast, no significant difference was found between secure attachment, insecure attachment, and neutral groups that were primed verbally.

#### Interpersonal Competence

The 2 × 3 ANCOVA for interpersonal competence showed that the main effect of attachment style was significant, *F*(1,392) = 3.53, *p* = 0.03, η^2^ = 0.02, but the main effect of priming modality was not, *F* < 0.08, *p* = 0.78, η^2^ = 0.00. Interpersonal competence scores were greater in the following order: secure attachment group (*M* = 98.52, *SD* = 14.15), neutral group (*M* = 97.53, *SD* = 13.32), and insecure attachment group (*M* = 94.14, *SD* = 17.65). As expected, there was a significant interaction between priming modality and attachment style, *F*(2,392) = 4.60, *p* = 0.01, η^2^ = 0.02. As displayed in Figure 2B, a Bonferroni *post hoc* analysis showed that interpersonal competence was greater for the group in which secure attachment was primed non-verbally (*M* = 99.86, *SD* = 14.98) than for the group in which insecure attachment was primed non-verbally (*M* = 90.94, *SD* = 19.99), *p* = 0.01. Also, interpersonal competence was greater for the non-verbal neutral group (*M* = 98.94, *SD* = 13.70) than for the group in which insecure attachment was primed non-verbally (*M* = 90.94, *SD* = 19.99), *p* = 0.03. In contrast, no significant difference was found between secure attachment, insecure attachment, and neutral groups that were primed verbally.

#### Positive Affect

The 2 × 3 ANCOVA for positive affect showed that the main effect of attachment style was significant, *F*(1,392) = 83.19*, p* = 0.00, η^2^ = 0.30, but the main effect of priming modality was not, *F* < 1.7, *p* = 0.20, η^2^ = 0.004. Positive affect was greater in the following order: secure attachment group (*M* = 28.36, *SD* = 8.02), neutral group (*M* = 23.39, *SD* = 6.25), and insecure attachment group (*M* = 16.73, *SD* = 8.50). As expected, there was a significant interaction between priming modality and attachment style, *F*(2,392) = 4.96, *p* = 0.01, η^2^ = 0.03. As displayed in Figure 2C, a Bonferroni *post hoc* analysis showed that for the non-verbal condition, positive affect was greater for the secure attachment group (*M* = 28.27, *SD* = 8.16) than for the neutral group (*M* = 24.21, *SD* = 6.31), *p* = 0.03, which in turn greater than for the insecure attachment group (*M* = 14.69, *SD* = 7.52), *p* = 0.00. In a similar way, for the verbal condition, positive affect was greater for the secure attachment group (*M* = 28.46, *SD* = 7.94) than for the neutral group (*M* = 22.64, *SD* = 6.14), *p* = 0.00, which in turn greater than for the insecure attachment group (*M* = 18.70, *SD* = 8.98), *p* = 0.00. However, the significant priming modality × attachment style interaction suggests that the effect of attachment style on positive affect appears prominent for the non-verbal priming condition as compared to that for the verbal priming condition.

In summary, for the non-verbal priming condition, the insecure attachment group reported lower self-esteem and interpersonal competence than the secure attachment group and the neutral group. In contrast, for the verbal priming condition, no significant differences in self-esteem and interpersonal competence were found between the secure attachment group, the insecure attachment group, and the neutral group. For both priming conditions, the insecure attachment group reported lower positive affect than the neutral group, which in turn lower than the secure attachment group. However, the differences in positive affect between the insecure attachment group and the other groups were greater for the non-verbal priming condition than for the verbal priming condition. Overall our results suggest that priming attachment non-verbally rather than verbally is more useful for observing the characteristics of each type of attachment.

## Discussion

Across two studies, we tested the effect of non-verbal priming on the activation of secure and insecure attachment styles. Specifically, the relative benefit of non-verbal priming compared to verbal priming was observed in the differences found in self-esteem, interpersonal competence, and positive affect according to attachment style.

In Study 1, through the use of scenario priming in the non-verbal condition, we emphasized the significant other’s gaze, voice, and touch to activate attachment styles. By revealing the relationship between secure attachment and various non-verbal cues that engage visual, tactile, and auditory senses, our results go one step beyond the previous finding that a single non-verbal cue—physical contact—activates secure attachment (Debrot et al., 2013; Jakubiak and Feeney, 2016a). However, in using this method the possibility arises that when a participant recollects the interaction with a significant other, the sense of attachment arises from a mix of non-verbal and verbal cues. Since memory is composed of both cues, it can be hard to distinguish between them.

To compensate for this possibility, in Study 2 attachment was primed using a standard method for comparing non-verbal and verbal cues: presenting photographs in the non-verbal condition and words that describe the photographs in the verbal condition (Snodgrass and Vanderwart, 1980). As a result, the group with secure attachment primed by non-verbal cues showed higher self-esteem, had more confidence in interpersonal relationships, and experienced greater positive affect than the group with insecure attachment primed by non-verbal cues. This finding is consistent with the results of previous research that used priming without distinguishing between non-verbal and verbal channels (Downey and Feldman, 1996; Murray et al., 2002; Pickett et al., 2004; Anthony et al., 2007). In contrast, differences between the groups that were primed verbally were either not significant or relatively smaller compared to the non-verbal condition. More specifically, if we consider the two attachment groups and the neutral group in the non-verbal priming condition, the insecure attachment group reported significantly lower self-esteem and interpersonal competence compared to the secure attachment group and the neutral group. In contrast, between the secure attachment group and the neutral group, no significant differences were found when measuring self-esteem and interpersonal competence. Why is the difference between the secure attachment group and neutral group relatively smaller compared to that between the insecure attachment group and neutral group? This may be attributable to the percentage of the attachment style in the general population. Several studies indicate that more than 60% of people have a secure attachment style (Campos et al., 1983; Hazan and Shaver, 1987; Collins and Read, 1990; Mikulincer, 1995). Based on this finding, it is plausible to assume that the neutral group was closer to the secure attachment group than to the insecure attachment group. On the other hand, positive affect, regardless of the priming condition, was greater in the following order: secure attachment group, neutral group, and insecure attachment group. This may be because the measurement variable is affect rather than thinking about oneself in terms of self-esteem and interpersonal competence. Sroufe and Waters (1977) stress the importance of emotions in attachment, because the goal of attachment is “felt security.” In addition, attachment is the primary experience in learning how to regulate emotions (Schore, 2003). Considering these points, positive affect seems to be even more connected with attachment than self-esteem and interpersonal competence, in which such beliefs as “My self-esteem is high” and “I can do well with my interpersonal relationships” are emphasized. As a result, it seems that attachment priming had more impact on affective than cognitive measurement. Future research may investigate the relationship between primed attachment styles and emotion, including positive and negative affect.

Also, why did a significant difference occur more frequently when the attachment was primed non-verbally than verbally? There are several possibilities. First, the vividness and intensity of attachment activated by non-verbal cues may have been higher and, in turn, better at evoking sensory experience than verbal cues (DeVries et al., 2003; Ditzen et al., 2008). In support of this, Willander and Larsson (2008) showed that participants in an odor imagery group who were asked to imagine the stimuli’s odor had more strongly activated sensory modalities—such as visual, olfactory, and auditory senses—than participants in a verbal group who were only presented with words like “tobacco,” “beer,” and “soap.” Similarly, Jakubiak and Feeney (2016b) found that imagining being touched buffered stress and pain more than verbal support. In our study, non-verbal and verbal stimuli constituted a between-subject variable; thus, it was not possible to explore how each individual experienced the intensity of schemas differently according to their activation under non-verbal or verbal conditions. In the case of respondents who scored at least four-points on the manipulation check, it was deemed that the relevant schema was activated and the analysis was conducted. However, this issue is worth considering in greater depth in a subsequent study.

Second, because of the nature of verbal cues, there may have been a relatively noticeable difference between the groups primed by non-verbal cues. For instance, when one actually receives or imagines the verbal support of a significant other in a stressful situation, one may worry about being evaluated by the other (Bolger and Amarel, 2007; Uchino, 2009; Feeney and Thrush, 2010; Taylor et al., 2010; Jakubiak and Feeney, 2016b). Verbal support focuses on one’s ability to address the stressor or problem (e.g., “It’s okay—you can do better next time”), which can tacitly pressure one into thinking, after being comforted, that one must perform well to maintain the approval of the attachment figure. Further, verbal support may threaten self-efficacy by being construed as support that is provided due to one’s vulnerability (Jakubiak and Feeney, 2016b). In contrast, non-verbal cues are used to convey valence without articulating a specific message. Therefore, these cues seldom lead to pressure or decreased self-efficacy. Perhaps due to these differences in the nature of verbal and non-verbal cues, secure attachment activated by verbal priming may not have as great an effect as secure attachment activated by non-verbal priming. However, it is still difficult to explain why the differences between verbally primed groups are smaller than those between non-verbally primed groups. Thus, we cannot assume that verbal priming is ineffective.

We do not mean to suggest that non-verbal cues provide the only access to the process of forming and activating attachment representations. Verbal and non-verbal cues are not separate in everyday life, because non-verbal behavior unconsciously gives rise to verbal expression, and verbal behavior unconsciously gives rise to non-verbal reactions (Knapp et al., 2013). Verbal and non-verbal cues can convey emotions, attitudes, personality traits, and reactions most effectively when combined to form a holistic interaction system. Therefore, we suggest that follow-up studies proceed from a comprehensive point of view that takes into account the nature of non-verbal cues.

The methodological limitations of this study are as follows. First, subsequent studies should pay more attention to measuring non-verbal cues. Study 1 used written instructions and guided imagery to prime non-verbal cues, which is an experimental method based on previous studies. During the priming procedure, however, there is a possibility that the non-verbal and verbal clues mixed. To overcome this limitation, in Study 2 we presented photographs and the words that describe them; this method is used frequently in non-verbal research. However, again, the instructions to participants had to use language. Also, although the photographs and words that are generally used in non-verbal research are concrete objects such as a table, spider, and bus (Snodgrass and Vanderwart, 1980), as an abstract concept, “attachment” is difficult to accurately match with photographs and their descriptions in words. In future research, it will be worth considering whether there is a method for priming with non-verbal stimuli by employing physiological and physical conditions and excluding the use of language.

Second, subsequent studies will require more fine-grained attention to insecure attachment, which can be further divided into avoidant and anxious attachment. However, in this study, attachment was divided into only two styles: secure and insecure. This was because it is an exploratory study of the importance of non-verbal cues in attachment, which has rarely been discussed; thus, we focused on secure and insecure attachment before examining them in detail. Moreover, if we consider the characteristics of insecure attachment, it would be difficult to divide it into avoidance attachment and anxiety attachment through the use of priming methods only. This is because both attachment styles arise from the same failure to form a secure attachment to primary caregivers, but the differences are found in the child’s temperament, which can be regarded as the participant’s temperament. The DSM-5 includes two disorders in relation to attachment: Reactive Attachment Disorder, which corresponds to avoidance attachment, and Disinhibited Social Engagement Disorder, which corresponds to anxiety attachment. Children with both of these disorders experience abusive or neglectful interactions with caregivers, but it is assumed that children exhibit different reactions and impairments due to differences in inherent temperament (American Psychiatric Association [APA], 2013; Gabbard, 2014). More specifically, children who have Reactive Attachment Disorder have an innately introverted and overly sensitive temperament, and react in an avoidant manner when they experience abusive or extremely unhealthy interactions with the caregiver. In contrast, children who exhibit Disinhibited Social Engagement Disorder are innately extroverted and temperamentally predisposed to seek stimuli; it is assumed that they react with excessive sociality and impulsive behavior (Lemelin et al., 2002; Zeanah and Gleason, 2010). Thus, priming alone—such as presenting an insecure attachment figure or evoking memories of abusive interactions—may limit the ability to subdivide insecure attachment. In future research, a nuanced research design that considers the temperament of each participant may be required.

Finally, the secure attachment activated in this study is a state attachment representation. Although individuals have secure and insecure attachment representations as traits, they can have also secure and insecure attachment representations as states according to priming or specific circumstances (Baldwin and Fehr, 1995; Baldwin et al., 1996). Therefore, further investigation is required to determine whether the same pattern of results is present in the case of trait attachment representations.

## Ethics Statement

This study was carried out in accordance with the recommendations of the Yonsei University Research Ethics Committee. The protocol was approved by the Yonsei University Institutional Review Board. Participants gave written informed consent in accordance with the Declaration of Helsinki.

## Author Contributions

SS and YS developed the study concept and design. SS collected the data and performed the data analysis under the supervision of YS. SS drafted the manuscript. JS provided critical revisions. All the authors approved the final version of the manuscript for submission.

## Conflict of Interest Statement

The authors declare that the research was conducted in the absence of any commercial or financial relationships that could be construed as a potential conflict of interest.

## Footnotes

1 A sample response by participants in the condition in which secure attachment was non-verbally primed is as follows: “When I was little, I hugged my mom, and took naps frequently. Through a window, the sunlight was coming in, and, largely, there was no conversation. With her eyes closed, my mom had fallen asleep. With my face buried against my mom’s body, either I had been smelling her body, which I had liked, or I had been touching her chest or belly. My mom had mainly slept while making low breathing sounds. Because the breathing sounds were in a rhythmic cycle, in many cases I, too, fell asleep. It was the most peaceful time in my life.” A sample response by participants in the condition in which insecurity was verbally primed is as follows: “When I was in my senior year of high school, because I was very sad, I telephoned home. I went up to the rooftop to make the call, because I was really sad. My mom, who had been listening for a long time, said, ‘So what?’ When I heard her words, I thought, ‘Ah, I am the only one in the world…’ After hearing her words I hung up the phone after saying, ‘Is that so? It must be so. . . . I will hang up, Mom.”’

## Appendix

**(1) Secure attachment scenario primed by non-verbal cues of significant others**

Call to mind a warm moment when you were with someone; recall where you were and what you were doing at that moment and what made you feel so warm. Perhaps the other person was extremely supportive, did not judge you, and accepted you just as you are. More specifically, recollect in detail what the non-verbal cues of the other person were like at that moment, including the facial expression, hand gestures, bodily posture, and voice tone. With this in mind, write at least 3–5 sentences to describe these non-verbal cues and the situation, along with your feelings at the time.

**(2) Insecure attachment scenario primed by non-verbal cues of significant others**

Call to mind a cold moment when you were with someone; recall where you were and what you were doing at that moment and as what made you feel discouraged, rejected, or afraid. Perhaps the other person was judgmental and was accepting of you only when you performed at or above a certain level. More specifically, recollect in detail what the non-verbal cues of the other person were like at that moment, including the facial expression, hand gestures, bodily posture, and voice tone. With this in mind, write at least 3–5 sentences to describe these non-verbal cues and the situation, along with your feelings at the time.

**(3) Secure and insecure attachment scenarios primed by verbal cues of significant others**

The secure attachment scenario used here is the same as that in (1), but instructions for the non-verbal cue portion were changed to “More specifically, what did the other person tell you at that moment? Recollect the content of his or her words in detail, and write at least 3–5 sentences to describe those words and the situation, along with your feelings at the time.” The insecure attachment scenario primed by verbal cues of significant others is the same as the insecure attachment scenario used in (2), but with the non-verbal cues changed to verbal cues, as shown above.
